# Formation of Cu*_x_*Au_1−_*_x_* phases by cold homogenization of Au/Cu nanocrystalline thin films

**DOI:** 10.3762/bjnano.5.162

**Published:** 2014-09-10

**Authors:** Alona Tynkova, Gabor L Katona, Gabor A Langer, Sergey I Sidorenko, Svetlana M Voloshko, Dezso L Beke

**Affiliations:** 1 National Technical University of Ukraine “Kiev Polytechnic Institute”, 37 Prospect Peremogy, 03056 Kiev, Ukraine; 2 Department of Solid State Physics, University of Debrecen, P.O. Box 2, 4010 Debrecen, Hungary

**Keywords:** Cu/Au, grain boundary diffusion, nanofilms of intermetallic compounds, secondary neutral mass spectrometry (SNMS), solid state reaction

## Abstract

It is shown, by using depth profiling with a secondary neutral mass spectrometer and structure investigations by XRD and TEM, that at low temperatures, at which the bulk diffusion is frozen, a complete homogenization can take place in the Cu/Au thin film system, which leads to formation of intermetallic phases. Different compounds can be formed depending on the initial thickness ratio. The process starts with grain boundary interdiffusion, which is followed by a formation of reaction layers at the grain boundaries that leads to the motion of the newly formed interfaces perpendicular to the grain boundary plane. Finally, the homogenization finishes when all the pure components have been consumed. The process is asymmetric: It is faster in the Au layer. In Au(25nm)/Cu(50nm) samples the final state is the ordered AuCu_3_ phase. Decrease of the film thicknesses, as expected, results in the acceleration of the process. It is also illustrated that changing the thickness ratio either a mixture of Cu-rich AuCu and AuCu_3_ phases (in Au(25nm)/Cu(25nm) sample), or a mixture of disordered Cu- as well as Au-rich solid solutions (in Au(25nm)/Cu(12nm) sample) can be produced. By using a simple model the interface velocity in both the Cu and Au layers were estimated from the linear increase of the average composition and its value is about two orders of magnitude larger in Au (ca. 10^−11^ m/s) than in Cu (ca. 10^−13^ m/s).

## Introduction

Solid-state reactions in nanostructured thin film systems are interesting and challenging not only from the point of view of pure fundamental research, but are also important for technological applications. Examples for the latter are the metallization of integrated circuits (the formation of a nanometric NiSi layer on the Si substrate [1–2]), or the production of thin chemically ordered FePt films for perpendicular magnetic data recording [3–4]. Regarding the basic understanding of such reactions the questions about the contributions of a fast mass transport along different grain boundaries (GBs, i.e., short circuits) can be mentioned; they can have an important effect on the entire intermixing process in nanocrystalline bi- or multilayers. In addition the GB diffusion coefficients can cover a range of several orders of magnitude, depending on the type of GB structure (low or high angle GBs [5], triple junctions [6]). Furthermore it can be observed at very low temperatures that the morphology of the formation and the growth of the new phase(s) can be different from the usual planar growth of a reaction layer [7–9]: The new phase(s) can be formed at grain boundaries, GBs, and can grow further by the motion of the new interfaces perpendicular to the original GB plane [10].

There are examples in the literature, in which the so-called “cold homogenization” was observed: Although the bulk diffusion processes were practically frozen in binary nanocrystalline couples even a complete intermixing of components leading to full homogenization was found [11–15]. Two reasons were mentioned as possible explanation for this phenomenon: i) diffusion-induced grain boundary motion (DIGM) and/or diffusion-induced re-crystallization (DIR), and ii) grain boundary motion during usual re-crystallization [11–12]. In the latter the alloying is the consequence of the alloyed zones left behind by re-crystallization during diffusion intermixing and as a result of grain growth the grain size should be increased. On the other hand during DIGM the composition behind the moving boundary can be about several tenth of an atomic fraction, i.e., the homogenization of a thin film with small grain size is also possible by this mechanism. During DIR, which is another manifestation of the stress relaxation caused by the initial inequality of the GB diffusion fluxes of the two components, new grains are formed, with a composition more discontinuously different from the surrounding grains as compared to DIGM.

In addition, it is difficult to make a distinction between DIR and DIGM experimentally [16]. DIR has mainly been investigated in binary systems with a wide mutual solubility range above either the miscibility gap or the critical temperature of ordering (e.g., in Cu/Pd [16], Au/Cu [15], Ag/Pd [7], Ni/Cu [17–19], NiPd [20]). Less works have been devoted to systems with reactive diffusion [21]. For instance in the Cu/Pd system [8,22] no reaction layers were detected at the original interface (see the transmission electron microscopy (TEM) image in Figure 9b of [8]) at 200 °C, but the selected-area diffraction patterns in TEM indicated the presence of the PdCu phase. After heat treatments at 260 °C, an extensive intermixing had taken place, accompanied by grain boundary migration, grain growth, and formation of CuPd and Cu_3_Pd phases. Similar results were obtained in Cu/Pd [23] Ni_2_Si/Si [24] and Fe/Pt [9] systems. In [10] these results were summarized and an interpretation based on the grain boundary diffusion induced reaction layer formation, GBDIREAC, was offered. Thus, it was proven that in binary systems with intermetallic layers not only a homogenization (by formation of solid solutions) but formation of compounds is also possible. In addition the morphology of the growing phases in such thin film couples can be different from the planar growth mode: Instead of nucleation and growth of the reaction layer at the initial interface, the reaction takes place in the GBs and the amount of the product phase grows by the motion of the formed new interfaces perpendicular to the GBs. Thus, the entire layer of the pure parent films can be consumed by interface diffusion driven interface motion and a fully homogeneous product layer can be obtained. Furthermore, in the first stage of such a process, assuming that the interface velocity is constant, the average composition in the center of the films should linearly increase with time (by gradually consuming the initial material of the grains) and the slope of this function is proportional to interface velocity [24–26].

In this study, similar processes are investigated in Au/Cu system at low temperatures. Note that in [7] this system was investigated in detail above the ordering temperature and comparative measurements were carried out only at 230 and 350 °C (below the ordering temperature). It was obtained that at higher temperatures the phase formation kinetics was very similar to the one obtained in Ag/Au system, when only disordered phases were formed. It was also concluded that in the Au/Cu system the main driving force was the chemical intermixing and the driving force to ordering gave only a minor contribution, i.e., the structural transformation was similar below and above the ordering temperature. The initial sharp planar interface still existed in the heat-treated samples and, similarly to the results of [8] in Cu/Pd, the early stages in Cu/Au could not be understood as a planar layer reaction. It was also observed that new grains were formed in the reaction zone (DIR). While in [7] the main phenomenon was the relaxation of mismatch stress (accumulated mainly by bulk diffusion above the ordering temperature), in this paper we will concentrate on processes at low temperatures, at which the bulk diffusion is completely frozen.

## Results

**Figure 1:**
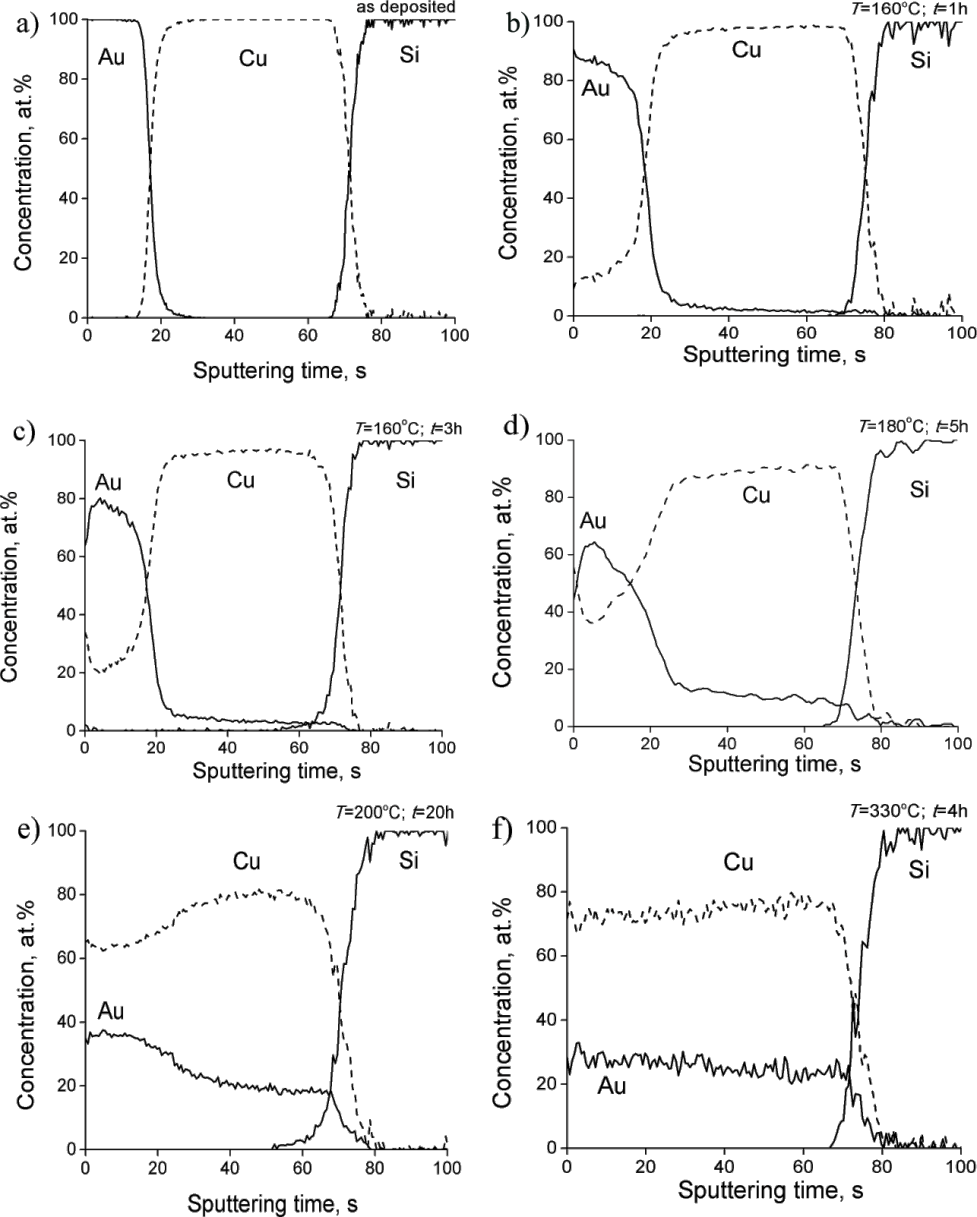
Concentration profiles of Au(25nm)/Cu(50nm) system a) as deposited sample and annealed b) at 160 °C for 1 h and c) 3 h, (d) 180 °C for 5 h, (e) 200 °C for 10 h, (f) 330 °C for 4 h.

The concentration profiles of Au(25nm)/Cu(50nm) samples annealed at different temperatures are shown in Figure 1. The presence of the smeared interface in the as-deposited sample can be explained by some initial surface roughness, diffusion during the sample preparation or instrumental effects of the sputter depth profiling [27–28]. It can be clearly seen that, during heat treatments the Cu penetration into the Au layer is more intensive than the Au penetration into the Cu layer. During annealing at 160 °C for 3 h, on the Au-side a significant intermixing occurs with Cu concentration up to 20 atom %, while on the Cu-side the concentration of Au atoms reaches only 7 atom % (Figure 1c). It can also be seen (see, e.g., Figure 1b and Figure 1c) that there are no plateaus on the composite profiles (as indications of formation of compound layers with planar interfaces in depth profiles taken, e.g., by SNMS) at shorter times, but there is a relatively high average composition of Cu inside the Au layer (Figure 1b) as well as of the Au composition inside the Cu layer (Figure 1d). In addition there is a minimum of the Cu profile inside the Au layer. These are the consequences of the GB mass transport along the GBs. The complete filling-up of grain boundaries, e.g., in Au would lead to a maximum average composition of about 7 atom % (since, with δ = 0.5 nm grain boundary thickness, 2δ/*d* is 0.066), and about 10% would be expected for this value in the Cu films, using the estimated average GB volume fraction calculated from the grain sizes (*d*_Au_ = 15 nm, *d*_Cu_ = 10 nm, see below). In Figure 1e the overall composition of the diffusing elements on both sides is rather high and cannot be simply explained by a filling-up of grain boundaries only, because the values obtained are larger than the one corresponding to the average value estimated from the volume fraction of the grain boundary area at the observed grain sizes.

It is noteworthy that the appearance of some Cu atoms at the topmost surface and the development of a minimum can be observed in the center of the Au layer (Figure 1c). It can be explained by the segregation of Cu [29] and/or by the coexistence of fast and slow diffusion boundaries (bimodal GB network) [25]. It was shown in [25] that in the latter case the GB diffusion starts along GBs with the largest diffusivities and there is only short penetration along GBs with small diffusivity values. At longer annealing times the GB penetration length is larger than the thickness of the film and the transported atoms spread out on the free surface forming a new source for diffusion along the still not filled GBs with smaller diffusivities. As a result a minimum in the average composition profile develops inside the film, closer to the free surface. Thus in our case the minimum of the Cu composition in the Au layer (Figure 1c) can also be a consequence of the bimodal GB structure. A complete homogenization of the system takes place both at low temperatures for longer annealing times (Figure 1e) and/or at higher temperatures (Figure 1f).

We would like to emphasize that we did not observe a reaction layer at the original interface in our depth profiles. This indicates that instead of nucleation of the product layer at the original interface, the new phase(s) formed in the whole volume of the films. In addition the compound phases have been formed without the participation of volume diffusion (according to bulk diffusion data the bulk diffusion penetration length is about 2.8 × 10^−11^ m in pure Cu at 250 °C for 30 min [30]). From Figure 1f it is clearly seen that the average compositions on both sides gradually leveled off and the result is the formation of an almost homogeneous layer with about 75 atom % of Cu and 25 atom % of Au composition.

**Figure 2:**
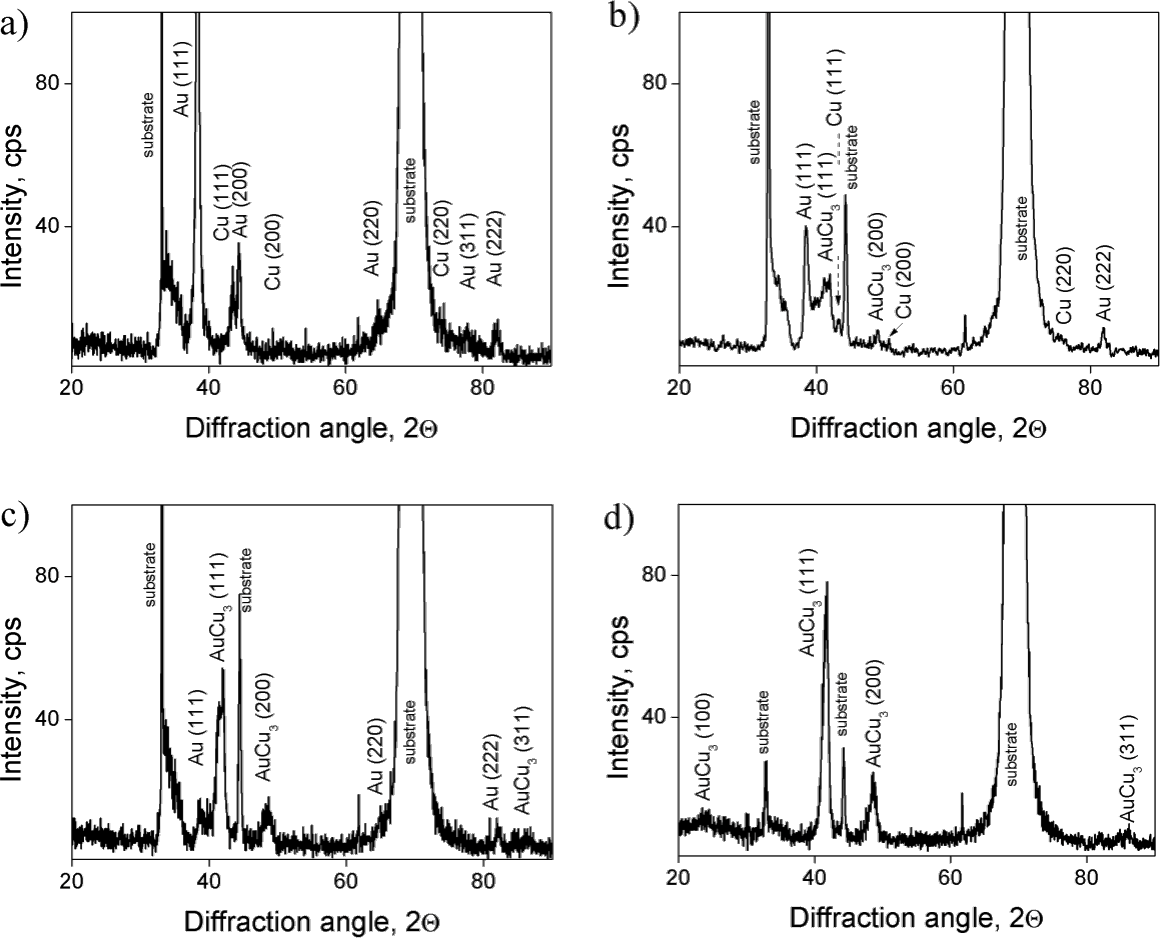
XRD θ–2θ patterns of Au(25nm)/Cu(50nm) samples a) as deposited, b) annealed at 180 °C for 5 h, c) for 10 h and d) at 200 °C for 44 h.

The XRD patterns of as deposited and annealed samples are shown in Figure 2. The weak reflection at 23.7°, belonging to a super lattice structure, indicates that the AuCu_3_ phase that is formed during the homogenization process (Figure 2d) is partially ordered. This is in agreement with SNMS data, indicating the presence of a slightly Cu rich AuCu_3_ phase.

The estimated grain sizes, *d* (from the full width at half maximum of the (111) peaks of Au and Cu by using the Debye–Scherrer formula [31–32]), are 15 nm for Au crystals and 10 nm in the case of Cu. After annealing at 180 °C for 5 h they decrease to *d* = 9 nm and *d* = 2 nm for Au and Cu, respectively (Figure 2b). The grain size of the newly formed AuCu_3_ phase (after 10 h of heat treatment at 180 °C) was estimated from the (111) peaks and 6 nm was obtained (Figure 2c). Regarding the reliability of the grain sizes estimated it is worthy of mention that calculations based on the Debye–Scherrer formula provide underestimated values for *d* [32], because of the fact that besides instrumental effects and grain size other factors (like inhomogeneous strain and crystal lattice imperfections) also contribute to the width of a diffraction peak.

**Figure 3:**
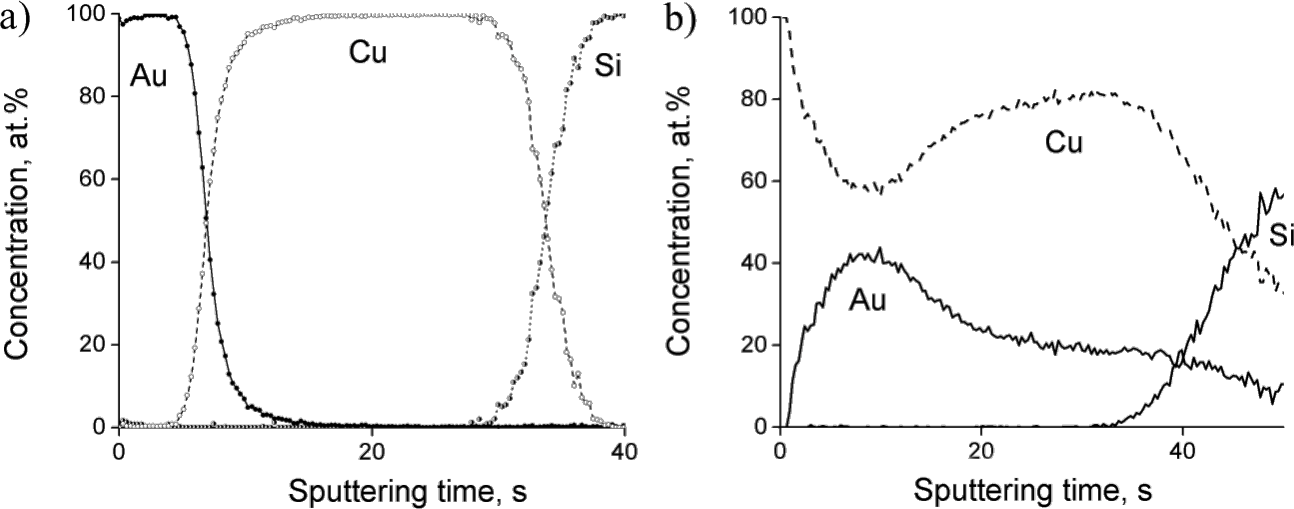
Concentration profiles of Au(10nm)/Cu(25nm) system a) as deposited and b) annealed at 180 °C for 5 h samples.

In order to investigate the effect of the individual thicknesses of the films, samples with thicknesses of 10 and 25 nm of Au and Cu, respectively, were also annealed under the same conditions. The concentration profiles are shown in Figure 3.

**Figure 4:**
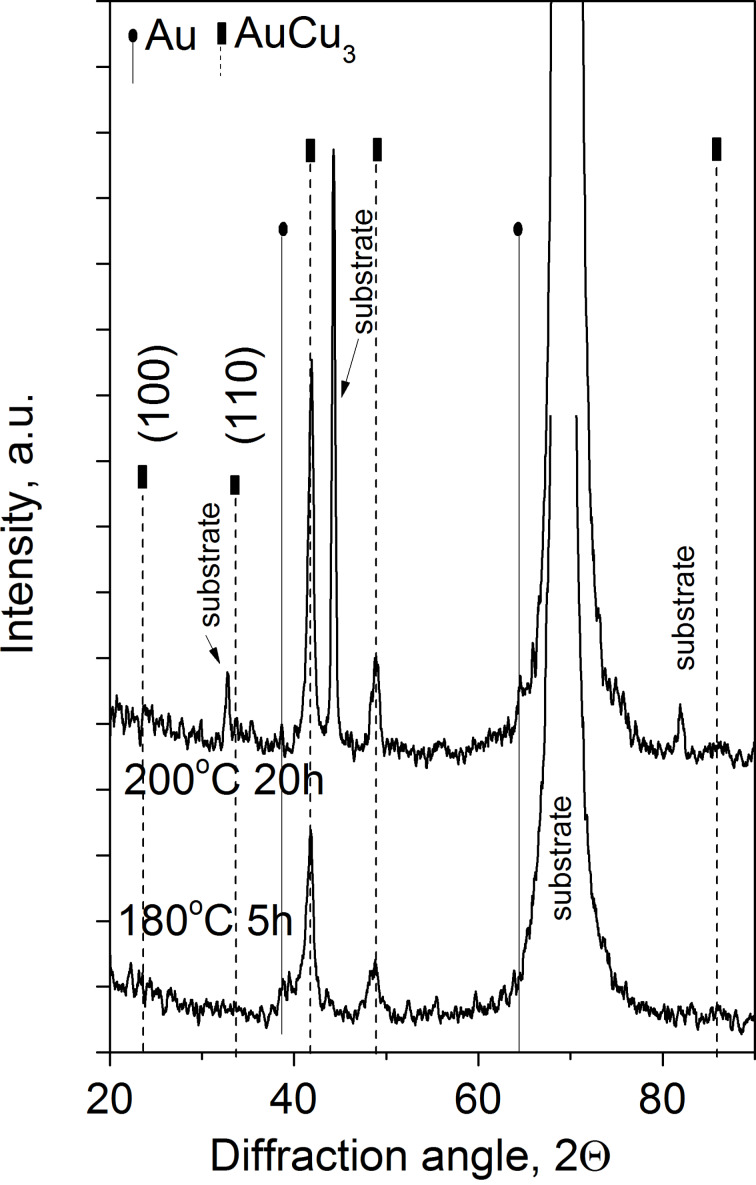
XRD θ–2θ patterns of Au(10nm)/Cu(25nm) annealed samples.

It can be seen that the process is quite similar to the samples with film thicknesses 25 and 50 nm (compare Figure 3b to Figure 1d) but the diffusion processes develop faster. Thus, already after 5 h of annealing at 180 °C the AuCu_3_ phase has been formed. The XRD patterns shown in Figure 4 also illustrate the formation of this AuCu_3_ phase. It is difficult to identify whether it is ordered or disordered: The expected positions of the super-lattice reflections (100) and (110) are indicated too.

**Figure 5:**
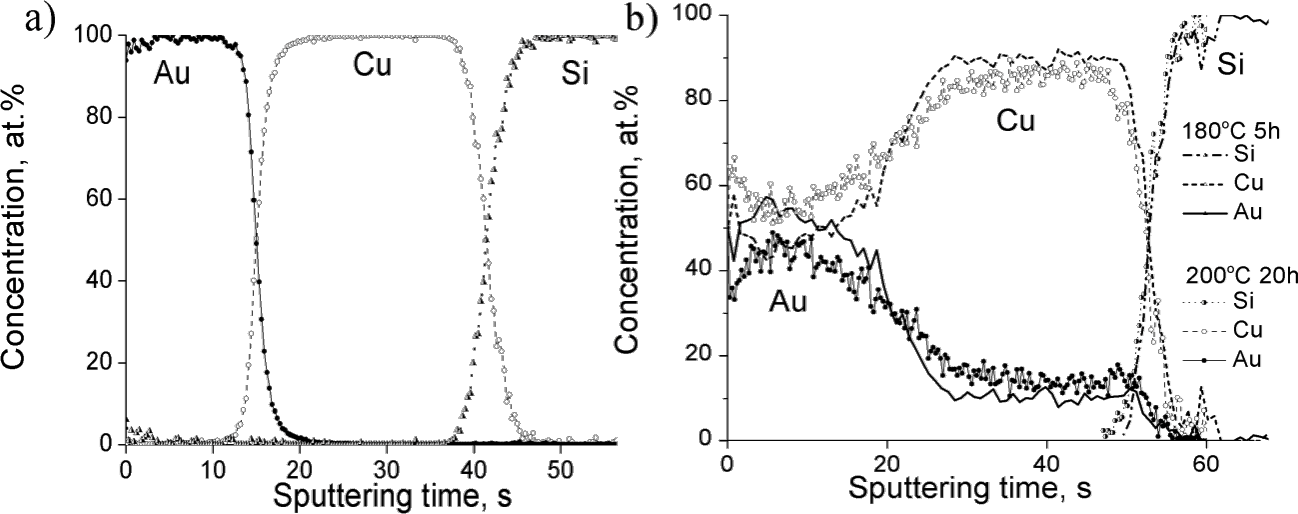
Concentration profiles of Au(25nm)/Cu(25nm) system a) as deposited sample and b) annealed samples.

In order to investigate the effect of the overall composition (i.e., the effect of the thickness ratio), measurements on samples Au(25nm)/Cu(25nm) and Au(25nm)/Cu(12nm) have also been carried out. It can be seen in Figure 5 that already after 5 h at 180 °C a homogeneous layer (of about the same thickness as the thickness of the original Au layer) has been formed with a 50/50 composition in the topmost layer in the place of gold. On the other hand, there is a considerable increase (up to about 10%) of the Au composition in the center of the Cu layer.

**Figure 6:**
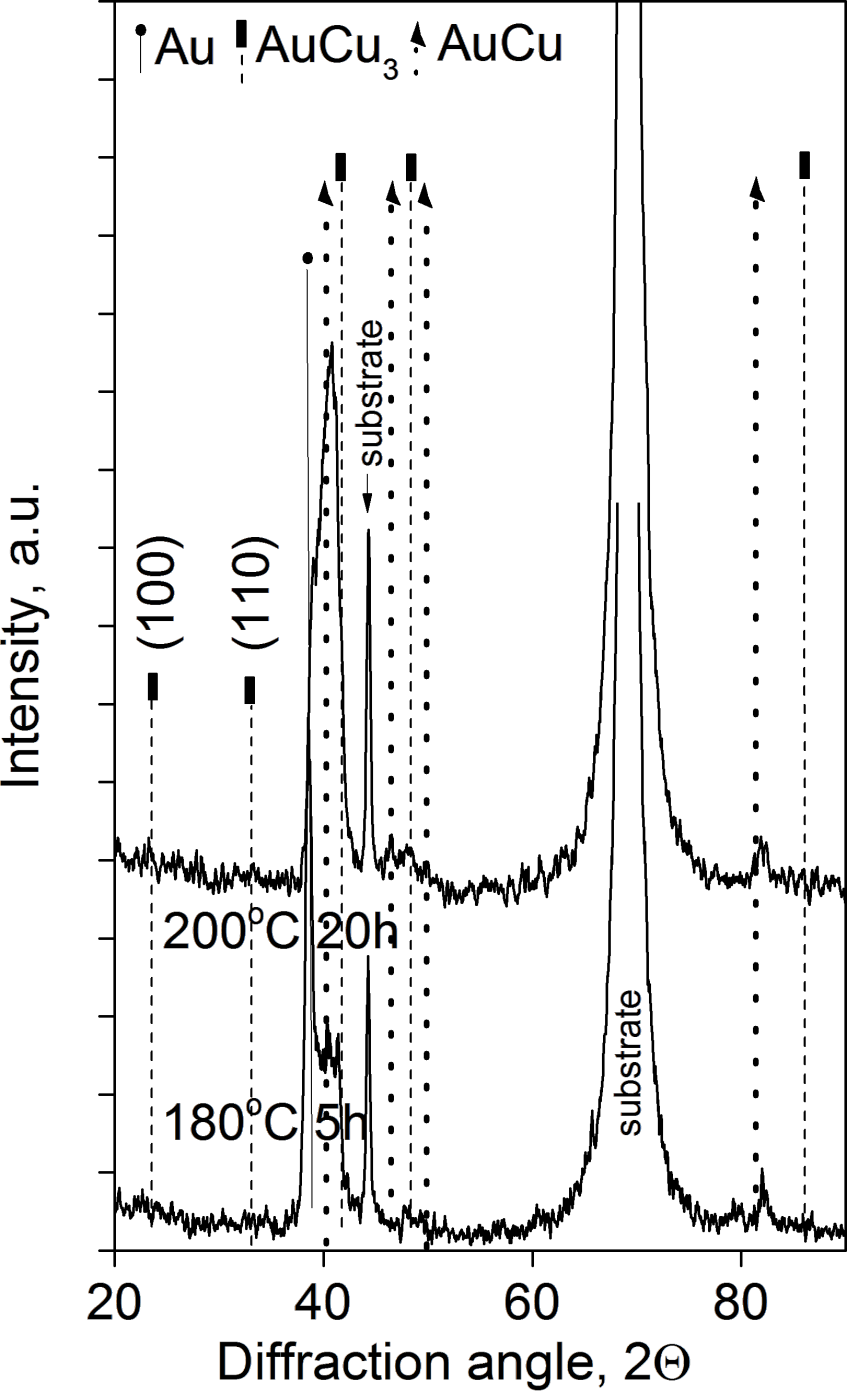
XRD θ–2θ patterns of Au(25nm)/Cu(25nm) annealed samples.

Figure 6 shows the θ–2θ XRD patterns of Au(25nm)/Cu(25nm) annealed samples. It can be seen that reflections of both AuCu and AuCu_3_ disordered phases can be observed already after annealing at 180 °C after 5 h. These results are in line with the profiles shown in Figure 5b: At longer annealing times the average composition of the Au inside the Cu film gradually increases and at 200 °C after 20 h the system seems to be a mixture of Cu-rich AuCu and AuCu_3_ phases.

**Figure 7:**
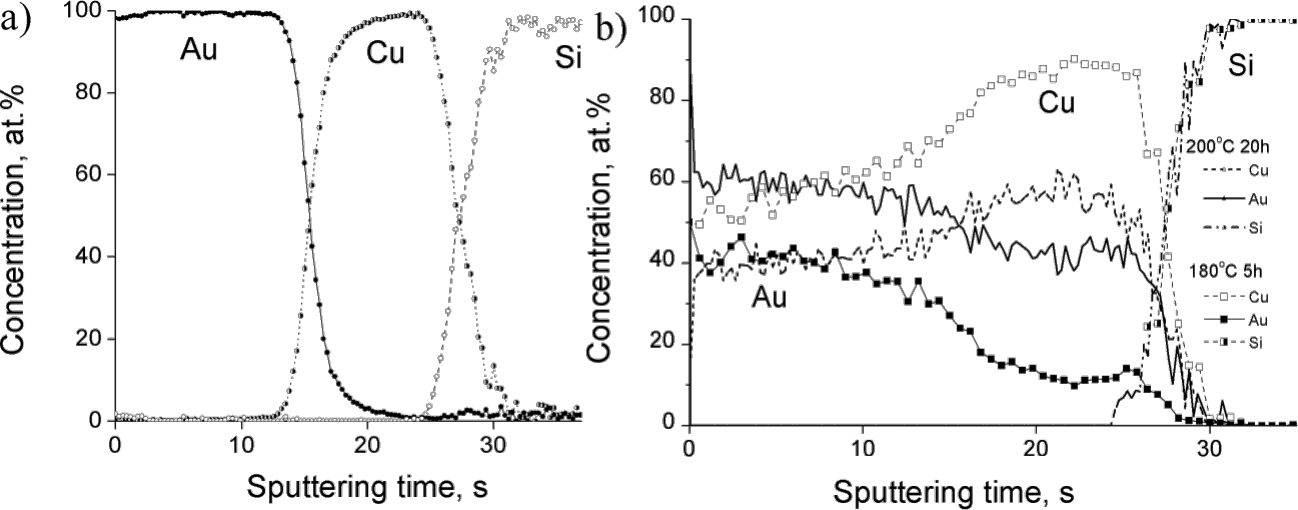
Concentration profiles of Au(25nm)/Cu(12nm) system a) as deposited sample and b) annealed samples.

In addition, Figure 7 illustrates that with a proper choice of the film ratio one can arrive at a mixture of Cu- and Au-rich AuCu solid solutions.

**Figure 8:**
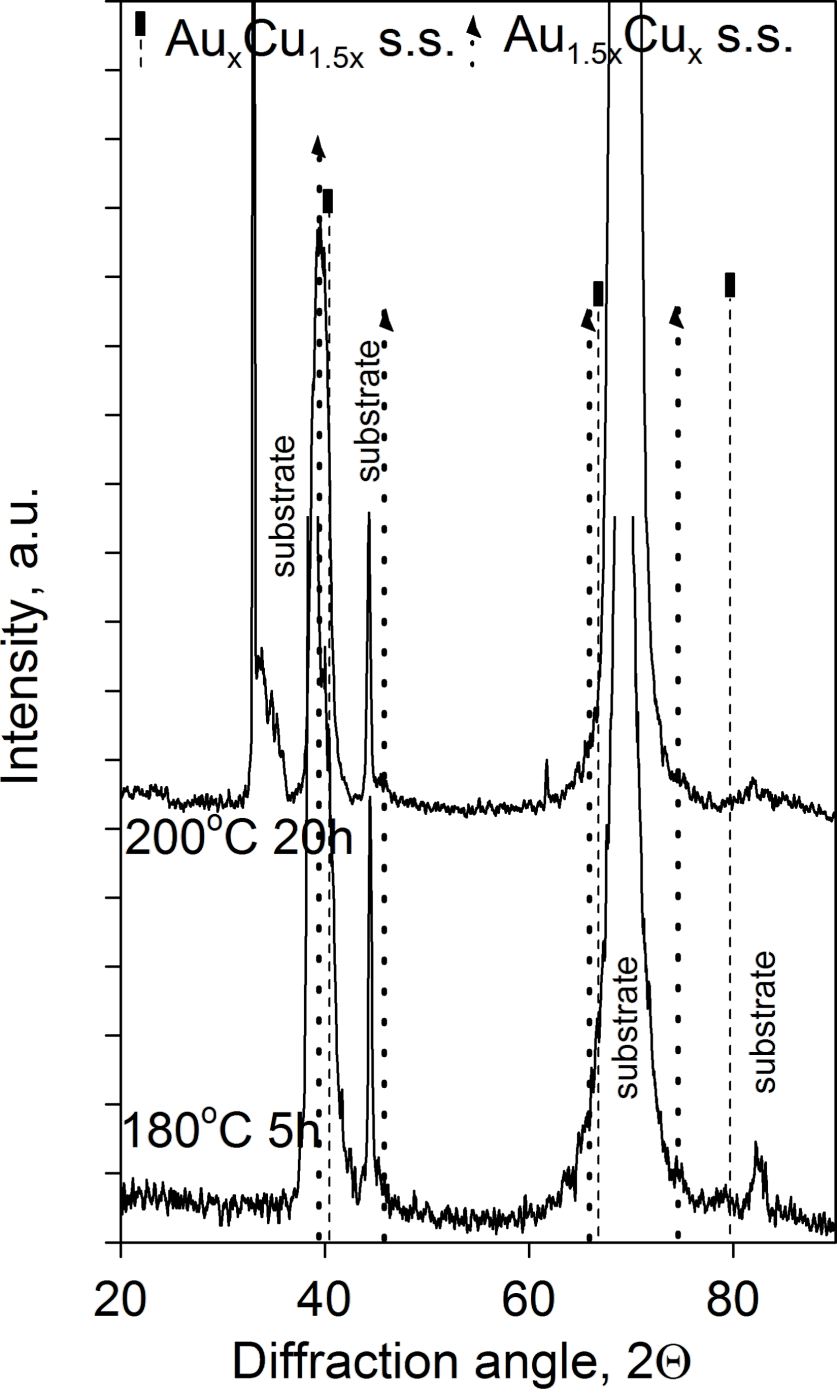
XRD θ–2θ patterns of Au(25nm)/Cu(12nm) annealed samples.

The XRD patterns shown in Figure 8 confirm this: Reflections of the disordered AuCu solid solutions can be identified. The vertical lines correspond to Au_1.5_*_x_*Cu*_x_* and Au*_x_*Cu_1.5_*_x_* solid solutions.

**Figure 9:**
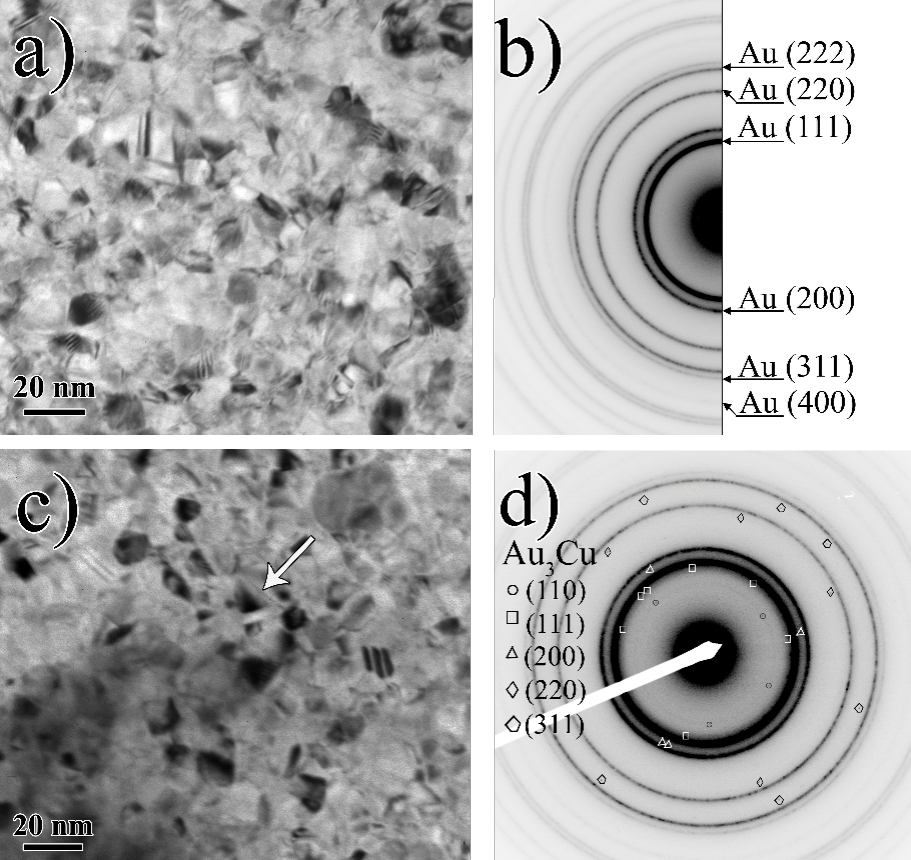
Bright field (top view) TEM images of Au(10nm)/Cu(15nm) bilayer a) as deposited and c) after 1 h of heat treatment at 160 °C. The arrow indicates the area of formation of a new phase. Selected area electron diffraction patterns of Au(10nm)/Cu(15nm) bilayer b) as deposited and d) after 1 h of heat treatment at 160 °C.

Figure 9 shows bright field (top view) TEM images and selected area electron diffraction patterns of as deposited and heat treated (for 1 h at 160 °C) Au(10nm)/Cu(15nm) bilayers, respectively. For TEM investigations the specimens were prepared by subsequent magnetron sputtering on monocrystalline sodium chloride substrates at room temperature. After the heat treatment the substrate was dissolved and the self-supporting film was used in TEM investigations. It can be seen that there is no detectable change in the grain size after the heat treatment, which is about 10 nm. The diffraction pattern of as deposited sample shows clear reflections from Au (Figure 9b) and after annealing additional diffraction peaks are found that correspond to the ordered Au_3_Cu phase (Figure 9d). In addition, the arrow in Figure 9c indicates a region of the formation of the reacted layer around a grain boundary; the indicated reflections pertain to this phase.

## Discussion

Our results indicate a special way of nucleation and growth of homogeneous reaction products in AuCu system. In accordance with the results of [7] in the same system the observations cannot be understood as a planar layer reaction: No continuous reaction layer formation was observed at the original interface. Instead homogenization of the initial pure layers can be characterized with a gradual increase of the composition in the center of the layers. Furthermore, we can conclude that all these phenomena should be the result of grain boundary transport as far as the bulk diffusion is negligible at such low temperatures. Thus the GB diffusion initiates the nucleation of the reaction product and sweeps the GBs perpendicular to the original surface and as a result, an alloyed zone remains behind the moving GBs. The obtained grain sizes favor this interpretation in contrast to the grain boundary motion during usual re-crystallization [11–12]. In the latter case, a grain growth should be observed at the same time as the homogenization. But our results, both obtained from XRD and TEM investigations, show that the average grain size either remained the same or even decreased after the heat treatments. Thus, the overall cold homogenization takes place through grain boundary diffusion induced grain boundary motion and a solid state reaction controlled by the interface diffusion along the newly formed interfaces. The XRD and TEM results confirmed the conclusions drawn from the SNMS depth profiles: Indeed the formation of reaction layers took place, and after longer annealing times even the super lattice reflections of the formed AuCu_3_ phase could be detected, indicating the ordering of this phase.

It is noteworthy that the above processes are interface/grain boundary diffusion controlled: After the formation of the new phase in the GBs, the process takes place through the atomic transport along the moving GB (like in the classical DIGM, when a solid solution is left behind) or along the interfaces of the new ordered phase (in this case the original GB is replaced by two new interfaces). In the latter case it is an interesting and open question whether both interfaces move or dominantly only one of these shifts: Direct in situ TEM investigations can help to clarify this point.

Nevertheless, by using the results of [24,26] we can estimate the velocity of the interfaces moving perpendicular to the initial grain boundary. According to this model one can assume that in thin films the grain size, *d*, is usually less than the film thickness, *H*. It is plausible to assume a spherical grain structure (2*R*_0_ = *d*) with a δ/2 thick spherical shell at the beginning (at *t* = 0), which grows with time by *vt*, where *v* is the constant interface velocity. Then the internal shell, in which the initial composition is *c*_0_, has the radius


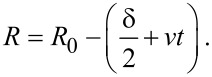


Assuming that the interface shift starts only after, e.g., Au GBs have been filled up to the equilibrium Cu composition of the growing phase, *c*_e_, then the average concentration, *c*, in the middle of the film can be given as

[1]
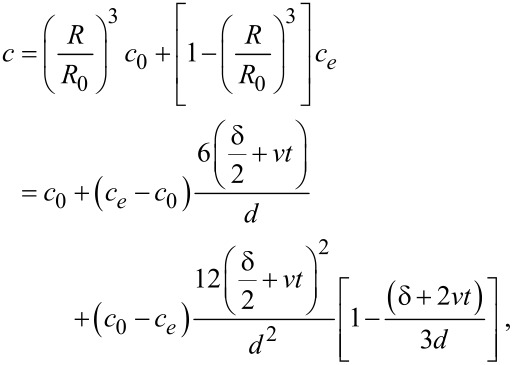


which, neglecting terms (δ + 2*vt*)/*d* on the third power, has the form, with *c*_e_ (in Au) = 0.5, *c*_e_ (in Cu) = 0.25 and *c*_0_ = 0:

[2]
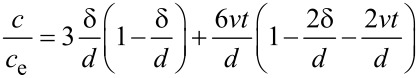


**Figure 10:**
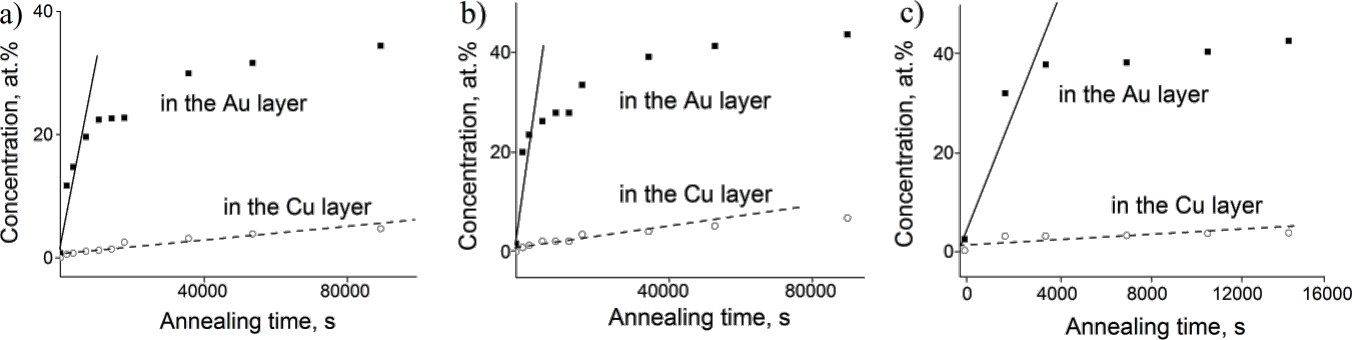
Dependence of the average concentration of elements on the annealing time at 150 °C in a) Au(25nm)/Cu(50nm), b) Au(25nm)/Cu(25nm) and c) Au(25nm)/Cu(12nm) systems.

At short times, the last term in the last bracket of Equation 2 can be neglected leading to a linear relation. Figure 10 shows the average composition inside the gold and copper layers as the function of the annealing time obtained after heat treatments at 150 °C: The first part is linear (the saturation at longer *t* values is due to finite size effects). From the linear initial part the values of the interface velocity can be obtained (Table 1).

**Table 1:** Calculated values for the velocity of moving interfaces.

	Au(25nm)/Cu(50nm)	Au(25nm)/Cu(25nm)	Au(25nm)/Cu(12nm)

in the Au layer	7 × 10^−12^ m/s	1.4 × 10^−11^ m/s	3 × 10^−11^ m/s
in the Cu layer	6 × 10^−14^ m/s	2 × 10^−13^ m/s	5 × 10^−13^ m/s

It can be seen that the interface velocity is about two orders of magnitude higher in Au than in Cu: This is plausible if we take into account a similar tendency in the grain boundary self-diffusion coefficients. In addition, our results offer an explanation for the linear growth kinetics in this regime: If the front velocity is constant, the amount of the product phase should grow linearly with time and the activation energy obtained from this part should be close to the activation energy of GB/interface diffusion [24,26].

## Conclusion

It is shown that at low temperatures, at which the bulk diffusion is frozen, an almost complete homogenization can take place in the Cu/Au thin film system, leading to the formation of intermetallic phases. It is illustrated that the process is based on grain boundary diffusion induced grain boundary motion and reaction layer formation, the process starts by grain boundary interdiffusion and after the filling-up of grain boundaries the reaction starts here. After the formation of the reaction zone (solid solution or ordered phase) the atomic transport along the original GB, or along the newly formed interfaces perpendicular to the grain boundary plane, results in the growth of the reacted material. Finally, the homogenization finishes when all the pure components have been consumed.

The initial part, in accordance with the well-known rule of thumb declaring that the diffusion is faster in the component with lower melting point, is asymmetric: The process is faster in the Au layer.

In Au(25nm)/Cu(50nm) samples, according to Figure 1f and Figure 2d, the final state is the ordered AuCu_3_ phase. Decreasing the film thicknesses (see the results obtained in Au(10nm)/Cu(25nm) and shown in Figure 3b and Figure 4) results, as expected, in the acceleration of the process.

It is illustrated that by changing the thickness ratio either a mixture of Cu-rich AuCu disordered and AuCu_3_ phases (see Figure 5b and Figure 6 for Au(25nm)/Cu(25nm) sample), or a mixture of disordered Cu- as well as Au-rich solid solutions (see Figure 7 and Figure 8) can be produced.

By using a simple model, we were able to estimate the interface velocity in both the Cu and Au layers from the linear increase of the average composition and, again in accordance with the above rule of thumb, its value is about two orders of magnitude larger in Au (of the order of 10^−11^ m/s) than in Cu (of the order of 10^−13^ m/s).

## Experimental

Au/Cu nanocrystalline thin films were prepared by DC magnetron sputtering onto (001)-oriented Si wafers with native SiO_2_ layer. The following bilayer samples were deposited: Au(25nm)/Cu(50nm), Au(25nm)/Cu(25nm), Au(25nm)/Cu(12nm), Au(10nm)/Cu(25nm) and Au(10nm)/Cu(15nm). During the deposition of metal layers the Si substrate was kept at room temperature and the Ar base pressure was set at 0.5 Pa. The rates of the deposition for Au and Cu layers were 0.85 nm/s and 0.5 nm/s, respectively.

The samples were annealed under vacuum (1 × 10^−4^ Pa) at temperatures ranging from 160 to 330 °C. The evolution of the intermixing process was studied over a time between 0.5 and 44 h.

The concentration profiles were measured by using a secondary neutral mass spectrometer (SPECS INA-X), that works with noble gas plasma and the bombarding ion current has an extremely high lateral homogeneity. The low bombarding energies (of the order of 100 eV) and the homogeneous plasma profile result in an outstanding depth resolution (smaller 2 nm). Details of the SNMS device and the profile evaluation can be found elsewhere [27–28].

The crystalline structure was examined by means of X-ray diffraction in θ–2θ scanning geometry while using Cu Kα radiation (Rigaku Ultima IV diffractometer) and by transmission electron microscopy (TEM, JEOL 2000FX-II).
